# Calcium Signaling in Cholangiocytes: Methods, Mechanisms, and Effects

**DOI:** 10.3390/ijms19123913

**Published:** 2018-12-06

**Authors:** Michele Angela Rodrigues, Dawidson Assis Gomes, Michael Harris Nathanson

**Affiliations:** 1 Section of Digestive Diseases, Department of Internal Medicine, Yale University School of Medicine, 333 Cedar Street, New Haven, CT 06520-8019, USA; michele.rodrigues@yale.edu (M.A.R.); dawidson.gomes@yale.edu (D.A.G.); 2 Department of Biochemistry and Immunology, Federal University of Minas Gerais. Av. Antônio Carlos, 6627, Belo Horizonte—MG 31270-901, Brazil

**Keywords:** Ca^2+^, inositol 1,4,5-trisphosphate (InsP_3_), inositol 1,4,5-trisphosphate receptors (ITPRs), cholangiocytes, biliary tree, secretion

## Abstract

Calcium (Ca^2+^) is a versatile second messenger that regulates a number of cellular processes in virtually every type of cell. The inositol 1,4,5-trisphosphate receptor (ITPR) is the only intracellular Ca^2+^ release channel in cholangiocytes, and is therefore responsible for Ca^2+^-mediated processes in these cells. This review will discuss the machinery responsible for Ca^2+^ signals in these cells, as well as experimental models used to investigate cholangiocyte Ca^2+^ signaling. We will also discuss the role of Ca^2+^ in the normal and abnormal regulation of secretion and apoptosis in cholangiocytes, two of the best characterized processes mediated by Ca^2+^ in this cell type.

## 1. Introduction

Intracellular Ca^2+^ controls a wide range of processes, including cell proliferation, apoptosis, and secretion [1,2,3,4]. The effects of Ca^2+^ signals have been extensively studied in hepatocytes, but less so in cholangiocytes [5]. Bile secretion is one of the primary functions of the liver and is the net result of bile formation by hepatocytes, followed by the conditioning of bile by cholangiocytes [5,6]. In both hepatocytes and cholangiocytes, Ca^2+^ signaling is mediated by inositol 1,4,5 trisphosphate (InsP_3_), which promotes Ca^2+^ release from the endoplasmic reticulum (ER) through binding to the inositol 1,4,5-trisphosphate receptor (ITPR) [7,8]. Although there are common mechanisms for generating ITPR-mediated Ca^2+^ signals among cell types, each cell type has distinct temporal and spatial patterns of Ca^2+^ signaling. Therefore, the way cells organize their Ca^2+^ machinery is crucial for the types of signals they can generate, and for how this second messenger regulates different cell functions within a cell. This review will discuss the organization of the Ca^2+^ toolkit that generates specific signals in cholangiocytes. The effects of various cholangiopathies on proteins that regulate Ca^2+^ signals will also be reviewed.

## 2. Biliary Tree Overview

Cholangiocytes constitute only 3–5% of the total population of nucleated cells in the liver [9]. The characteristics of cholangiocytes vary depending on their anatomic location within the biliary tree. The bile canaliculus that continues into the Canal of Hering is the ductule-canalicular junction. This is the transition point for the biliary lumen, where it becomes lined by cholangiocytes rather than hepatocytes. The biliary epithelium is also partially formed by undifferentiated hepatic progenitor cells [10]. These cells are the resident stem cell compartments in the liver and are capable of differentiating into either cholangiocytes or hepatocytes [11]. The human intrahepatic bile duct increases in size as it moves towards the common bile duct, from cholangioles (<15 μm) to small bile ducts (15–300 μm) to large bile ducts (300–800 μm) [9]. The intrahepatic biliary ductal system in rodents has a heterogeneous morphology and has been classified according to whether cholangiocytes are small (<15 μm) or large (>15 μm) in diameter [12,13]. Large and small cholangiocytes also have functional heterogeneity, which includes differences such as absorptive, secretory, proliferative, and apoptotic ability [14,15]. Cholangiocytes play a role in the modification of alkalinity and the composition of primary bile by the secretion of chloride (Cl^−^) and bicarbonate (HCO_3_^−^) [6] and by absorbing bile salts, amino acids, and glucose. Large versus small cholangiocytes appear to participate differentially in this [15]. Extending from the apical plasma membrane into the bile duct lumen are the cholangiocyte cilia, which detect changes in bile flow, osmolality, and composition [16]. Cilia maintain a separate, higher Ca^2+^ concentration than the cytosol, and ciliary stimuli can affect the Ca^2+^ concentration either within the cilia or in the rest of the cytosol, or both [17,18,19,20,21]. Components of the cilia that relate to Ca^2+^ signaling include the Ca^2+^ channels PKD1L1, PKD2L1, and the transient receptor potential vanilloid subfamily 4 (TRPV4) channels, as well as the mechanosensors PKD1 and PKD2 [21,22,23]. PKD1L1 associates with PKD2L1 via coiled-coil domains to act as a ciliary Ca^2+^ channel [17,18], while TRPV4 has been implicated in the signal transduction of osmotic stimuli [19]. Abnormalities in ciliary structure and functions are responsible for acquired and inherited liver diseases, such as polycystic liver disease. Ciliary structural defects and integrated sensory/transducing functions appear to be related and result in decreased intracellular Ca^2+^ and increased cAMP [24,25]. These signaling alterations, in turn, result in cholangiocyte hyperproliferation, altered fluid secretion and absorption, and abnormal cell-matrix interactions, which could contribute to altered structure and function [16]. The pharmacological activation of TRPV4 has been proposed as a way to restore the reduced intracellular Ca^2+^ levels seen in polycystic liver disease, and thereby decrease proliferation and cyst growth [20].

## 3. Experimental Models to Study Signaling in Cholangiocytes

Various approaches have been developed to study cholangiocyte physiology. Perhaps the first model of secretory physiology in bile ducts was the isolated bile duct unit (IBDU), which consists of small segments of freshly isolated bile ducts that became sealed in short-term culture and permits the assessment of secretion by monitoring the rate of expansion of the enclosed lumen [26,27,28,29]. This model continues to be useful, but a modified version was subsequently developed in which the bile duct segment is micro-dissected and then cannulated at both ends to permit direct access, manipulation, and monitoring of both the basolateral and apical (luminal) sides of the bile duct segment [30,31,32]. This modification requires significant technical expertise but maintains the complexity and function of bile ducts. This model has been used, for example, to show that Ca^2+^ signals and their effects on HCO_3_^−^ secretion depend in part on the differential subcellular localization of ITPR isoforms [30]. These findings also relate to the molecular basis of various cholestatic disorders [33]. In cholangiocytes, the only intracellular Ca^2+^ release channel is the ITPR [27] and micro-dissected, microperfused bile ducts were used to show that the loss of expression of the type 3 isoform in particular is part of a final common pathway in cholestatic conditions [30,33]. Both cAMP- and Ca^2+^-mediated pathways are important for ductular HCO_3_^−^ secretion, and it has been thought that the two pathways are largely distinct. However, this experimental model has also been used to show that the two pathways converge, and that secretin/cAMP/cystic fibrosis transmembrane conductance regulator (CFTR)-mediated HCO_3_^−^ secretion depends on cytosolic calcium and on ITPR3 in particular [30]. 

Primary cholangiocytes isolated from the liver and its related cell lines are the most widely used experimental models. There are very few cholangiocyte cell lines available and primary liver cells rapidly dedifferentiate in culture, which substantially limits their usefulness. Because cholangiocytes are only a small fraction of the total population of nucleated cells in the liver, it is important to carefully isolate them from all other liver cell types. The cholangiocyte isolation protocol has been refined over the years to obtain a pure cholangiocyte cell fraction from total liver, and further recent progress permits cells to be obtained with proper polarity [34,35,36,37,38,39]. To obtain proper two-dimensional (2D) or three-dimensional (3D) cultures, it is typically necessary to use a Percoll gradient and monoclonal antibodies, such as anti-Ep-CAM (clone HEA125), to isolate the cells based on size, density, and specific membrane components. Pure cholangiocytes are generally plated on collagen-coated cell culture plasticware and cultured with a special media formulation to maintain their phenotype. It is also possible to have cells with normal polarity if the cells are plated on transwell inserts with a semipermeable membrane [40]. When this method is used, it is possible for cells to maintain the proper epithelial barrier function, apical junctions, and cilia. The isolation and characterization of primary cilia from cholangiocytes has also been reported [41], which should provide an additional tool to investigate the role of primary cilia in normal and pathological conditions.

Genetic engineering tools have been a powerful way to study liver disease. These include methods such as differentiating pluripotent stem cells into cholangiocyte-like cells, which display functional and structural similarities to bile duct cells [42]. Pluripotent stem cells can differentiate into a range of cell types and display a robust ability to proliferate, which therefore represents an alternative and reproducible source of cell differentiation of therapeutic interest [42,43]. Another promising model is the use of induced pluripotent stem cells (iPSCs), which can provide a source of cells derived from patients that can be differentiated into somatic cells of interest. Those cells retain the genetic background of the cell donor, which makes them suitable for disease modeling [25]. Some studies have shown that it is possible to differentiate patient-derived iPSCs into liver epithelial cells and that those cells can reproduce the phenotype of the genetic disease of origin [25,44,45,46]. This includes a recently modified protocol for the differentiation of iPSCs into cholangiocytes as a model to study the cystic fibrosis transmembrane conductance regulator (CFTR), which is the channel mutated in cystic fibrosis, a common cause of cholangiopathy [25]. Such experimental models have improved our knowledge about the mechanisms that regulate cholangiocyte signal transduction, proliferation, apoptosis, secretion, and transport, along with corresponding alterations that lead to diseases of the biliary tract.

## 4. Regulation of Cholangiocyte Secretory Activity by Calcium Signaling

**Figure 1:**
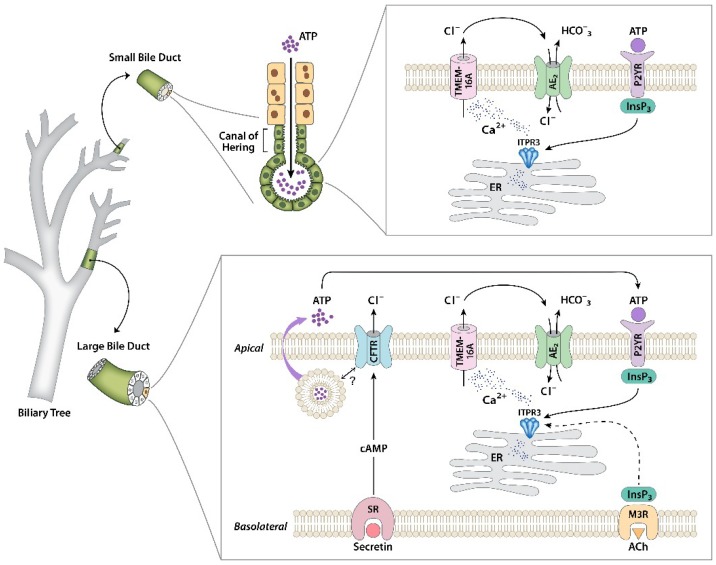
Regulation of bicarbonate secretion in cholangiocytes. Separate signaling pathways that regulate secretion have been identified in large and small cholangiocytes. In large cholangiocytes, secretin receptors (SRs) on the basolateral membrane link to formation of cAMP, which leads to the activation of cystic fibrosis transmembrane conductance regulators (CFTRs), causing apical Cl^−^ efflux. This also stimulates exocytic release of ATP into the ductular lumen, through a mechanism that has not yet been identified. Luminal ATP then binds to apical P2Y receptors to stimulate intracellular Ca^2+^ release via inositol 1,4,5-trisphosphate receptor isoform 3 (ITPR3), which in turn activates Cl^−^ secretion through TMEM16A in the apical membrane. The resulting Cl^−^ gradient across the apical membrane drives the AE2 Cl^−^/HCO_3_^−^ exchanger, resulting in net HCO_3_^−^ secretion. This pathway can also be activated directly by biliary ATP secreted from upstream hepatocytes. Alternatively, inositol 1,4,5-trisphosphate (InsP3) formed from the stimulation of the M3 muscarinic receptor can stimulate secretion, although there is some evidence that this may act through Ca^2+^ released from ITPR1 and ITPR2 rather than ITPR3. Small cholangiocytes lack SRs and CFTRs, but have the same apical calcium signaling machinery to link to HCO_3_^−^ secretion that is found in large cholangiocytes. Figure modified in part from References [9,30].

Several pathways are involved in increasing Ca^2+^ in the cytosol and in other subcellular compartments in cholangiocytes. For example, experiments using isolated primary mouse cholangiocytes, intrahepatic bile duct units, microdissected bile duct segments, and isolated, bivascularly perfused rat livers demonstrate that the stimulation of muscarinic receptors promotes secretion [6,30,47]. Specifically, acetylcholine (ACh) binds to M3 muscarinic receptors at the basolateral membrane to release InsP_3_ and diacylglycerol (DAG) via hydrolyses of phosphatidylinositol 4,5-bisphosphate (PIP_2_) by phospholipase C (PLC). InsP_3_ diffuses into the cytosol and binds to its receptor localized at the ER to promote the release of Ca^2+^ into the cytosol. Similarly, P2Y purinergic receptors (P2YR) on either the apical or basolateral membrane bind to ATP or UTP, leading to InsP_3_ dependent intracellular Ca^2+^ release [32]. The endogenous source of luminal ATP is not entirely clear. Hepatocytes are able to secrete ATP into bile, and this can be enhanced by certain bile acids [48,49]. Cholangiocytes can also release ATP into the ductular lumen [30,47]. This appears to depend on CFTRs, but there is also evidence that this ATP is released via a classical exocytosis pathway [50]. Studies using both rodent and human biliary epithelium demonstrated that stimulation with ursodeoxycholic acid (UDCA) or tauroursodeoxycholic acid (TUDCA) leads to increased exocytosis, with associated ATP release, increased Ca^2+^, Cl^−^ permeability, and transepithelial secretion. An integrated view of cholangiocyte secretory mechanisms is summarized in Figure 1.

Cholangiocytes express all three ITPR isoforms, and these are the only intracellular Ca^2+^ release channels in this cell type [27,33,51]. The ITPR isoform 3 (ITPR3) is the most heavily expressed isoform and is concentrated apically [27]. This subcellular distribution is found in both rodent and human cholangiocytes [27,33,52]. Ca^2+^ signals originate in the apical region of polarized epithelia because ITPR expression is most concentrated there [53,54], even though the specific isoform that is concentrated apically varies among cell types [53,54,55,56]. The channel open probability of ITPR3 displays a sigmoidal dependency on Ca^2+^ [57]. Consequently, Ca^2+^ signaling via this isoform may permit relatively high (up to 10 μM) Ca^2+^ concentrations to be attained in the apical region, which is important for the local activation of adaptor proteins from the snare complex [58] as well as the regulation of membrane fusion events that are relevant for the insertion of membrane transporters [59,60]. 

There are two parallel signaling pathways that regulate HCO_3_^−^ secretion in bile ducts, which are mediated by either Ca^2+^ or cAMP. Studies using isolated bile duct units and isolated cholangiocytes suggest that the stimulation of Cl^−^ secretion by cAMP occurs via the cystic fibrosis transmembrane conductance regulator (CFTR), and the secretion of HCO_3_^−^ occurs via AE2, an associated Cl^−^/HCO_3_^−^ exchanger [7,51,61]. In a normal intact liver, this mechanism of ductular secretion has been demonstrated by bivascular perfusion via the hepatic artery and the portal vein in isolated liver [6]. This approach was useful because the blood supply reaches cholangiocytes via the hepatic artery. These studies showed that ductular HCO_3_^−^ secretion is induced by ACh, which is a Ca^2+^ agonist, and that this event depends on both Cl^−^ channels and Cl^−^/HCO_3_^−^ exchange. However, cAMP-mediated HCO_3_^−^ secretion also depends upon Cl^−^ channels [6,62]. In cholangiocytes, cAMP-dependent fluid secretion is typically activated by secretin [63,64]. Secretin receptors (SRs) are expressed at the basolateral membrane and, upon secretin binding, cause intracellular cAMP formation. Protein kinase A (PKA) is activated by its second messenger, which then activates/phosphorylates CFTRs at the apical membrane, stimulating Cl^−^ secretion into the ductular lumen [65,66]. The efflux of Cl^−^ establishes a driving force for HCO_3_^−^ secretion by anion exchanger 2 (AE2) activation, causing the alkalization of bile via the SLC4A2 gene [65]. Cholangiocytes also express an apical Ca^2+^-activated Cl^−^ channel, which is the product of the transmembrane member 16A gene (TMEM16A) [50,67]. TMEM16A is also the bile acid-induced Cl^−^ secretion target in cholangiocytes in both rodents and humans [50]. 

## 5. Alterations in Ca^2+^ Signaling in Cholangiopathies

Ca^2+^ signaling machinery is altered in cholestatic disorders, which contributes to their pathophysiology [5,33,52,68]. The expression of ITPR3 is decreased in cholangiocytes from patients with sclerosing cholangitis, primary biliary cholangitis, benign or malignant biliary obstruction, biliary atresia, sepsis, and alcoholic hepatitis [33,68]. Specific knockdown of ITPR3 in cholangiocytes is sufficient to impair ductular HCO_3_^−^ secretion, which suggests that the loss of ITPR3 in human disorders contributes to cholestasis [30]. In this model, Ca^2+^ signals are impaired or absent [30]. Similarly, selective loss of bile duct ITPR3 expression is observed in animals subjected to lipopolysaccharide (LPS) injection or common bile duct ligation, which are accepted models of ductular cholestasis [33]. Several different mechanisms have been identified as responsible for the loss of ITPR3 from bile ducts in human diseases, and these mechanisms may be disease-specific. For example, LPS binds to TLR4 on cholangiocytes, which then activates NF-κB [52]. In turn, NF-κB binds to the ITPR3 promoter to decrease its expression in cholangiocytes. This mechanism is responsible for the loss of ITPR3 in patients with sepsis-associated cholestasis or severe alcoholic hepatitis [52]. Interestingly, in CFTR-defective cholangiocytes, Src tyrosine kinase self-activates and phosphorylates TLR4, also resulting in the activation of NF-κB and increased pro-inflammatory cytokine production in response to LPS [40]. The inhibition of Src furthermore attenuates endotoxin-induced biliary damage and inflammation in CFTR-knockout (CFTR-KO) mice [40]. However, it is not yet known whether ITPR3 expression is decreased in CFTR-defective mice. The transcription factor nuclear factor, erythroid 2-like 2 (NRF2), which is activated by oxidative stress, also can transcriptionally regulate ITPR3 expression [68]. This mechanism is at least partly responsible for the loss of ITPR3 seen in a variety of cholangiopathic disorders, including sclerosing cholangitis, primary biliary cholangitis, biliary obstruction, and biliary atresia [68], but not sepsis or alcoholic hepatitis [52]. Finally, miR-506 also inhibits ITPR3 expression in cholangiocytes, and this contributes to their loss in patients with primary biliary cholangitis [69]. These various mechanisms likely reflect direct actions on cholangiocytes rather than nonspecific effects resulting from peri-portal inflammation because ITPR3 expression is normal in the bile ducts of patients infected with Hepatitis C virus and these patients do not have clinical evidence of cholestasis, despite the presence of portal inflammation [33].

Because ductular cholestasis results from impaired Ca^2+^ signaling in cholangiocytes, the stimulation of Ca^2+^ signals may be a useful strategy to treat cholestasis. Indeed, the hydrophilic bile acid UDCA is of proven efficacy for treating a number of cholestatic liver diseases involving the bile ducts [70,71]. Several lines of evidence suggest that enhanced Ca^2+^ signaling mediates the cytoprotective effects of UDCA and its taurine conjugate TUDCA [47,48,72]. UDCA stimulates ATP secretion from hepatocytes into bile [48], which may promote bile flow and HCO_3_^−^ secretion by the paracrine activation of bile duct epithelial P2Y receptors, with subsequent activation of the Ca^2+^-dependent Cl^−^ channel TMEM16 and then AE2 [50,67]. Furthermore, UDCA can stimulate ductular secretion more directly by inducing cholangiocytes to release ATP by a CFTR-dependent mechanism, resulting in autocrine stimulation of P2Y receptors and then the Ca^2+^-dependent secretion of HCO_3_^−^, again involving TMEM16a and AE2 [30,47,50]. Thus, the stimulation of Ca^2+^ signals in cholangiocytes can be used as a strategy for the treatment of cholestatic disorders. 

## 6. Apoptosis in Cholangiocytes

Apoptosis in cholangiocytes occurs under both normal and pathological conditions and is regulated in part through Ca^2+^ signaling pathways. For example, during ductal development, apoptosis is a normal phenomenon that permits the regression of the ductal plate [14,73]. Aberrant ductal proliferation is reversed by apoptotic mechanisms in the setting of transient biliary obstruction [74]. It is also implicated in cholestatic liver diseases such as Primary Biliary Cholangitis (PBC), Primary Sclerosing Cholangitis (PSC), and biliary atresia, and has been described in rodent models of cholestatic liver disease [75,76,77].

Apoptosis depends on mitochondrial permeabilization, which results from excess mitochondrial Ca^2+^ signaling [78,79,80]. This is due to the transmission of Ca^2+^ from the ER to the mitochondria via ITPRs that are localized to specialized regions of the ER in proximity to the mitochondria [78,79,80,81]. There is functional evidence that each of the three ITPR isoforms may contribute to mitochondrial Ca^2+^ signaling and apoptosis [4]. However, when all three isoforms are expressed, then ITPR3 appears to colocalize most with the mitochondria and contribute most to mitochondrial Ca^2+^ signals and apoptosis [4], suggesting that it has the highest affinity for the ER–mitochondrial interface. On the other hand, ITPR1 is the only isoform that has been shown to physically reside in the ER–mitochondrial junction, where grp75 has been identified as the linker protein that attaches it to voltage-dependent anion channel (VDAC) in the outer mitochondrial membrane [82]. Moreover, it has been established that ITPR1 is the isoform that colocalizes with the mitochondria and is responsible for mitochondrial Ca^2+^ signaling in hepatocytes [83]. Other factors contribute to apoptosis as well, some of which also relate to mitochondrial Ca^2+^ signaling. Anti-apoptotic Bcl-2 family members such as Bcl-xl inhibit apoptosis via ITPR-mediated Ca^2+^ release by reducing ITPR expression [84]. Bcl-2 also contributes to Ca^2+^ leakage from ER [85]. One important Bcl-2 family member expressed in cholangiocytes is Mcl-1 [86], which inhibits apoptosis by attenuating mitochondrial Ca^2+^ signaling, even though Mcl-1 does not affect either ITPR expression or ER Ca^2+^ stores [3]. Because ITPR3 may be the most effective isoform to form signaling microdomains with the mitochondria [4], and the loss of ITPR3 makes cells resistant to apoptosis [87,88], it has been hypothesized that enhanced degradation of ITPR3 is a premalignant change. Because this isoform is mostly concentrated in the apical region of the cholangiocytes [27], it is not known whether apical ITPR3 also is associated with a sub-population of the mitochondria in cholangiocytes, or whether a separate, smaller sub-population of ITPR3 serves this role. Alternatively, there are small pools of ITPR1 and ITPR2 that are distributed throughout cholangiocytes [27], so it is possible that one or both of these isoforms selectively couples to mitochondria to control apoptosis, similar to the role of ITPR1 in hepatocytes [83]. 

## 7. Conclusions

Historically, bile ducts were thought to be little more than passive conduits to move canalicular bile from hepatocytes to the duodenum. Due in part to the availability of a variety of experimental models, it is now understood that cholangiocytes play an important role in the conditioning of bile, including its alkalinization. Furthermore, there is now a detailed understanding of the mechanisms involved in the regulation of ductular bile secretion, and in the molecular pathophysiology responsible for a variety of cholangiopathies. The identification of these new pathways has the potential to lead to the development of new therapeutic strategies.

## References

[1] Berridge M.J., Bootman M.D., Roderick H.L. (2003). Calcium signalling: Dynamics, homeostasis and remodelling. Nat. Rev. Mol. Cell Biol..

[2] Rodrigues M.A., Gomes D.A., Leite M.F., Grant W., Zhang L., Lam W., Cheng Y.C., Bennett A.M., Nathanson M.H. (2007). Nucleoplasmic calcium is required for cell proliferation. J. Biol. Chem..

[3] Minagawa N., Kruglov E.A., Dranoff J.A., Robert M.E., Gores G.J., Nathanson M.H. (2005). The anti-apoptotic protein Mcl-1 inhibits mitochondrial Ca^2+^ signals. J. Biol. Chem..

[4] Gomes D.A., Thompson M., Souto N.C., Goes T.S., Goes A.M., Rodrigues M.A., Gomez M.V., Nathanson M.H., Leite M.F. (2005). The type III inositol 1,4,5-trisphosphate receptor preferentially transmits apoptotic Ca^2+^ signals into mitochondria. J. Biol. Chem..

[5] Trampert D.C., Nathanson M.H. (2018). Regulation of bile secretion by calcium signaling in health and disease. Biochim. Biophys. Acta Mol. Cell Res..

[6] Hirata K., Nathanson M.H. (2001). Bile duct epithelia regulate biliary bicarbonate excretion in normal rat liver. Gastroenterology.

[7] Amaya M.J., Nathanson M.H. (2014). Calcium signaling and the secretory activity of bile duct epithelia. Cell Calcium.

[8] Amaya M.J., Nathanson M.H. (2013). Calcium signaling in the liver. Compr. Physiol..

[9] Lazaridis K.N., Strazzabosco M., Larusso N.F. (2004). The cholangiopathies: Disorders of biliary epithelia. Gastroenterology.

[10] Spee B., Carpino G., Schotanus B.A., Katoonizadeh A., Vander Borght S., Gaudio E., Roskams T. (2010). Characterisation of the liver progenitor cell niche in liver diseases: Potential involvement of Wnt and Notch signalling. Gut.

[11] Itoh T., Miyajima A. (2014). Liver regeneration by stem/progenitor cells. Hepatology.

[12] Alpini G., Glaser S., Robertson W., Phinizy J., Rodgers R., Caligiuri A., LeSage G. (1997). Bile acids stimulate proliferative and secretory events in large but not small cholangiocytes. Am. J. Physiol. Liver Physiol..

[13] Alpini G., Roberts S., Kuntz S.M., Ueno Y., Gubba S., Podila P.V., LeSage G., LaRusso N.F. (1996). Morphological, molecular, and functional heterogeneity of cholangiocytes from normal rat liver. Gastroenterology.

[14] Cheung A.C., Lorenzo Pisarello M.J., LaRusso N.F. (2018). Pathobiology of biliary epithelia. Biochim. Biophys. Acta Mol. Basis Dis..

[15] Marzioni M.M., Glaser S.S., Francis H., Phinizy J.L., LeSage G., Alpini G. (2002). Functional heterogeneity of cholangiocytes. Semin. Liver Dis..

[16] Masyuk A.I., Masyuk T.V., LaRusso N.F. (2008). Cholangiocyte primary cilia in liver health and disease. Dev. Dyn..

[17] Delling M., Decaen P.G., Doerner J.F., Febvay S., Clapham D.E. (2013). Primary cilia are specialized calcium signalling organelles. Nature.

[18] Decaen P.G., Delling M., Vien T.N., Clapham D.E. (2013). Direct recording and molecular identification of the calcium channel of primary cilia. Nature.

[19] Gradilone S.A., Masyuk A.I., Splinter P.L., Banales J.M., Huang B.Q., Tietz P.S., Masyuk T.V., LaRusso N.F. (2007). Cholangiocyte cilia express TRPV4 and detect changes in luminal tonicity inducing bicarbonate secretion. Proc. Natl. Acad. Sci..

[20] Gradilone S.A., Masyuk T.V., Huang B.Q., Banales J.M., Lehmann G.L., Radtke B.N., Stroope A., Masyuk A.I., Splinter P.L., LaRusso N.F. (2010). Activation of Trpv4 Reduces the Hyperproliferative Phenotype of Cystic Cholangiocytes From an Animal Model of ARPKD. Gastroenterology.

[21] Masyuk A.I., Gradilone S.A., LaRusso N.F. (2014). Calcium Signaling in Cilia and Ciliary-Mediated Intracellular Calcium Signaling: Are They Independent or Coordinated Molecular Events?. Hepatology.

[22] Lorenzo I.M., Liedtke W., Sanderson M.J., Valverde M.A. (2008). TRPV4 channel participates in receptor-operated calcium entry and ciliary beat frequency regulation in mouse airway epithelial cells. Proc. Natl. Acad. Sci..

[23] Köttgen M., Buchholz B., Garcia-Gonzalez M.A., Kotsis F., Fu X., Doerken M., Boehlke C., Steffl D., Tauber R., Wegierski T. (2008). TRPP2 and TRPV4 form a polymodal sensory channel complex. J. Cell Biol..

[24] Spirli C., Mariotti V., Villani A., Fabris L., Fiorotto R., Strazzabosco M. (2017). Adenylyl cyclase 5 links changes in calcium homeostasis to cAMP-dependent cyst growth in polycystic liver disease. J. Hepatol..

[25] Fiorotto R., Amenduni M., Mariotti V., Fabris L., Spirli C., Strazzabosco M. (2018). Src kinase inhibition reduces inflammatory and cytoskeletal changes in ΔF508 human cholangiocytes and improves cystic fibrosis transmembrane conductance regulator correctors efficacy. Hepatology.

[26] Nathanson M.H., Burgstahler A.D., Mennone A., Boyer J.L. (1996). Characterization of cytosolic Ca^2+^ signaling in rat bile duct epithelia. Am. J. Physiol..

[27] Hirata K., Dufour J.-F., Shibao K., Knickelbein R., O’Neill A.F., Bode H.-P., Cassio D., St-Pierre M.V., Larusso N.F., Leite M.F. (2002). Regulation of Ca^2+^ signaling in rat bile duct epithelia by inositol 1,4,5-trisphosphate receptor isoforms. Hepatology.

[28] Mennone A., Alvaro D., Cho W., Boyer J.L. (1995). Isolation of small polarized bile duct units. Proc. Natl. Acad. Sci. USA.

[29] Spirlì C., Nathanson M.H., Fiorotto R., Duner E., Denson L.A., Sanz J.M., Di Virgilio F., Okolicsanyi L., Casagrande F., Strazzabosco M. (2001). Proinflammatory Cytokines Inhibit Secretion in Rat Bile Duct Epithelium. Gastroenterology.

[30] Minagawa N., Nagata J., Shibao K., Masyuk A.I., Gomes D.A., Rodrigues M.A., Lesage G., Akiba Y., Kaunitz J.D., Ehrlich B.E. (2007). Cyclic AMP Regulates Bicarbonate Secretion in Cholangiocytes Through Release of ATP Into Bile. Gastroenterology.

[31] Masyuk A.I., Gong A.Y., Kip S., Burke M.J., LaRusso N.F. (2000). Perfused rat intrahepatic bile ducts secrete and absorb water, solute, and ions. Gastroenterology.

[32] Dranoff J.A., Masyuk A.I., Kruglov E.A., Larusso N.F., Nathanson M.H. (2001). Polarized expression and function of P2Y ATP receptors in rat bile duct epithelia. Am. J. Physiol. Gastrointest. Liver Physiol..

[33] Shibao K., Hirata K., Robert M.E., Nathanson M.H. (5085). Loss of Inositol 1,4,5-Triphosphate Receptors From Bile Duct pithelia Is a Common Event in Cholestasis. Gastroenterology.

[34] Joplin R., Strain A.J., Neuberger J.M. (1989). Immuno-isolation and culture of biliary epithelial cells from normal human liver. In Vitro Cell. Dev. Biol..

[35] Joplin R., Strain A.J., Neuberger J.M. (1990). Biliary Epithelial Cells From the Liver of Patients With Primary Biliary Cirrhosis : Isolation, Characterization, and Short-Term Culture. J. Pathol..

[36] Ishii M., Vroman B., LaRusso N.F. (1989). Isolation and Morphologic Cells From Normal Rat Liver. Gastroenterology.

[37] Vroman B., LaRusso N.F. (1996). Development and characterization of polarized primary cultures of rat intrahepatic bile duct epithelial cells. Lab Invest..

[38] Banales J.M., Sáez E., Uriz M., Sarvide S., Urribarri A.D., Splinter P., Tietz Bogert P.S., Bujanda L., Prieto J., Medina J.F., LaRusso N.F. (2012). Up-regulation of microRNA 506 leads to decreased Cl-/HCO_3_^-^ anion exchanger 2 expression in biliary epithelium of patients with primary biliary cirrhosis. Hepatology.

[39] Spirli C., Okolicsanyi S., Fiorotto R., Fabris L., Cadamuro M., Lecchi S., Tian X., Somlo S., Strazzabosco M. (2010). ERK1/2-Dependent Vascular Endothelial Growth Factor Signaling Sustains Cyst Growth in Polycystin-2 Defective Mice. Gastroenterology.

[40] Fiorotto R., Villani A., Kourtidis A., Scirpo R., Amenduni M., Geibel P.J., Cadamuro M., Spirli C., Anastasiadis P.Z., Strazzabosco M. (2016). The cystic fibrosis transmembrane conductance regulator controls biliary epithelial inflammation and permeability by regulating Src tyrosine kinase activity. Hepatology.

[41] Huang B.Q., Masyuk T.V., Muff M.A., Tietz P.S., Masyuk A.I., LaRusso N.F. (2006). Isolation and characterization of cholangiocyte primary cilia. AJP Gastrointest. Liver Physiol..

[42] Dianat N., Dubois-Pot-Schneider H., Steichen C., Desterke C., Leclerc P., Raveux A., Combettes L., Weber A., Corlu A., Dubart-Kupperschmitt A. (2014). Generation of functional cholangiocyte-like cells from human pluripotent stem cells and HepaRG cells. Hepatology.

[43] Dianat N., Steichen C., Vallier L., Weber A., Dubart-Kupperschmitt A. (2013). Human Pluripotent Stem Cells for Modelling Human Liver Diseases and Cell Therapy. Curr. Gene Ther..

[44] Ogawa M., Ogawa S., Bear C.E., Ahmadi S., Chin S., Li B., Grompe M., Keller G., Kamath B.M., Ghanekar A. (2015). Directed differentiation of cholangiocytes from human pluripotent stem cells. Nat. Biotechnol..

[45] Sampaziotis F., De Brito M.C., Madrigal P., Bertero A., Saeb-Parsy K., Soares F.A.C., Schrumpf E., Melum E., Karlsen T.H., Bradley J.A. (2015). Cholangiocytes derived from human induced pluripotent stem cells for disease modeling and drug validation. Nat. Biotechnol..

[46] Ghanekar A., Kamath B.M. (2016). Cholangiocytes derived from induced pluripotent stem cells for disease modeling. Curr. Opin. Gastroenterol..

[47] Fiorotto R., Spirlì C., Fabris L., Cadamuro M., Okolicsanyi L., Strazzabosco M. (2007). Ursodeoxycholic Acid Stimulates Cholangiocyte Fluid Secretion in Mice via CFTR-Dependent ATP Secretion. Gastroenterology.

[48] Nathanson M.H., Burgstahler A.D., Masyuk A., Larusso N.F. (2001). Stimulation of ATP secretion in the liver by therapeutic bile acids. Biochem. J..

[49] Schlosser S.F., Burgstahler A.D., Nathanson M.H. (1996). Isolated rat hepatocytes can signal to other hepatocytes and bile duct cells by release of nucleotides. Proc. Natl. Acad. Sci. USA.

[50] Li Q., Dutta A., Kresge C., Bugde A., Feranchak A.P. (2018). Bile acids stimulate cholangiocyte fluid secretion by activation of transmembrane member 16A Cl−channels. Hepatology.

[51] Pusl T., Nathanson M.H. (2004). The role of inositol 1,4,5-trisphosphate receptors in the regulation of bile secretion in health and disease. Biochem. Biophys. Res. Commun..

[52] Franca A., Filho A.C.M.L., Guerra M.T., Weerachayaphorn J., Santos M.L. dos, Njei B., Robert M., Lima C.X., Vidigal P.V.T., Banales J.M., Ananthanarayanam M. (2018). Effects of endotoxin on type 3 inositol 1,4,5-trisphosphate receptor in human cholangiocytes. Hepatology.

[53] Nathanson M.H., Fallon M.B., Padfield P.J., Maranto A.R. (1994). Localization of the type 3 inositol 1,4,5-Trisphosphate receptor in the Ca^2+^ wave trigger zone of pancreatic acinar cells. J. Biol. Chem..

[54] Nagata J., Guerra M.T., Shugrue C.A., Gomes D.A., Nagata N., Nathanson M.H. (2007). Lipid Rafts Establish Calcium Waves in Hepatocytes. Gastroenterology.

[55] Amaya M.J., Oliveira A.G., Schroeder L.K., Allgeyer E.S., Bewersdorf J., Nathanson M.H. (2014). Apical localization of inositol 1,4,5-trisphosphate receptors is independent of extended synaptotagmins in hepatocytes. PLoS ONE.

[56] Hirata K., Nathanson M.H., Burgstahler A.D., Okazaki K., Mattei E., Sears M.L. (1999). Relationship between Inositol 1,4,5-Trisphosphate Receptor Isofors and Subcellular Ca^2+^ Signaling Patterns in Nonpigmented Ciliary Epithelia. Invest. Ophthalmol. Vis. Sci..

[57] Hagar R.E., Burgstahler A.D., Nathanson M.H., Ehrlich B.E. (1998). Type III InsP3 receptor channel stays open in the presence of increased calcium. Nature.

[58] Fernández-Chacón R., Königstorfer A., Gerber S.H., García J., Matos M.F., Stevens C.F., Brose N., Rizo J., Rosenmund C., Südhof T.C. (2001). Synaptotagmin I functions as a calcium regulator of release probability. Nature.

[59] Hu K., Carroll J., Fedorovich S., Rickman C., Sukhodub A., Davietov B. (2002). Vesicular restriction of synaptobrevin suggests a role for calcium in membrane fusion. Nature.

[60] Söllner T., Bennett M.K., Whiteheart S.W., Scheller R.H., Rothman J.E. (1993). A protein assembly-disassembly pathway in vitro that may correspond to sequential steps of synaptic vesicle docking, activation, and fusion. Cell.

[61] Guerra M.T., Nathanson M.H. (2015). Calcium signaling and secretion in cholangiocytes. Pancreatology.

[62] Minagawa N., Ehrlich B.E., Nathanson M.H. (2006). Calcium signaling in cholangiocytes. World J. Gastroenterol..

[63] Boyer J.L. (1996). Bile duct epithelium: Frontiers in transport physiology. Am. J. Physiol..

[64] Alpini G., Phinizy J.L., Glaser S., Francis H., Benedetti A., Marucci L., LeSage G. (2003). Development and characterization of secretin-stimulated secretion of cultured rat cholangiocytes. Am. J. Physiol. Gastrointest. Liver Physiol..

[65] Kanno N., LeSage G., Glaser S., Alpini G. (2001). Regulation of cholangiocyte bicarbonate secretion. Am. J. Physiol. Gastrointest. Liver Physiol..

[66] Fouassier L., Duan C., Feranchak A.P., Yun C.H.C., Sutherland E., Simon F., Fitz J.G., Doctor R.B. (2001). Ezrin-radixin-moesin-binding phosphoprotein 50 is expressed at the apical membrane of rat liver epithelia. Hepatology.

[67] Dutta A.K., Khimji A.K., Kresge C., Bugde A., Dougherty M., Esser V., Ueno Y., Glaser S.S., Alpini G., Rockey D.C. (2011). Identification and functional characterization of TMEM16A, a Ca^2+^-activated Cl-channel activated by extracellular nucleotides, in biliary epithelium. J. Biol. Chem..

[68] Weerachayaphorn J., Amaya M., Spirli C., Chansela P., Mitchell K., Ananthanarayanan M., Nathanson M. (2015). Nuclear Factor Erythroid 2-like 2 Regulates Expression of Inositol 1,4,5-trisphosphate Receptor, Type 3 and Calcium Signaling in Cholangiocytes. Gastroenterology.

[69] Ananthanarayanan M., Banales J.M., Guerra M.T., Spirli C., Munoz-Garrido P., Mitchell-Richards K., Tafur D., Saez E., Nathanson M.H. (2015). Post-translational regulation of the type III inositol 1,4,5-trisphosphate receptor by miRNA-506. J. Biol. Chem..

[70] Paumgartner G., Beuers U. (2002). Ursodeoxycholic acid in cholestatic liver disease: Mechanisms of action and therapeutic use revisited. Hepatology.

[71] Poupon R.E., Poupon R., Balkau B. (1994). Ursodiol for the long-term treatment of primary biliary cirrhosis. N. Engl. J. Med..

[72] Marzioni M., Francis H., Benedetti A., Ueno Y., Fava G., Venter J., Reichenbach R., Mancino M.G., Summers R., Alpini G. (2006). Ca^2+^-dependent cytoprotective effects of ursodeoxycholic and tauroursodeoxycholic acid on the biliary epithelium in a rat model of cholestasis and loss of bile ducts. Am. J. Pathol..

[73] Terada T., Nakanuma Y. (1995). Detection of apoptosis and expression of apoptosis-related proteins during human intrahepatic bile duct development. Am. J. Pathol..

[74] Bhathal P.S., Gall J.A. (1985). Deletion of hyperplastic biliary epithelial cells by apoptosis following removal of the proliferative stimulus. Liver.

[75] Kremer A.E., Rust C., Eichhorn P., Beuers U., Holdenrieder S. (2009). Immune-mediated liver diseases: Programmed cell death ligands and circulating apoptotic markers. Expert Rev. Mol. Diagn..

[76] Erickson N., Mohanty S.K., Shivakumar P., Sabla G., Chakraborty R., Bezerra J.A. (2008). Temporal-spatial activation of apoptosis and epithelial injury in murine experimental biliary atresia. Hepatology.

[77] Takeda K., Kojima Y., Ikejima K., Harada K., Yamashina S., Okumura K., Aoyama T., Frese S., Ikeda H., Haynes N.M. (2008). Death receptor 5 mediated-apoptosis contributes to cholestatic liver disease. Proc. Natl. Acad. Sci. USA.

[78] Szalai G., Krishnamurhy R., Hajnóczky G. (1999). Apoptosis driven by IP3 -linked mitochondrial calcium signals. EMBO J..

[79] Csordás G., Thomas A.P., Hajnóczky G. (1999). Quasi-synaptic calcium signal transmission between endoplasmic reticulum and mitochondria. EMBO J..

[80] Ichas F., Jouaville L.S., Mazat J.P. (1997). Mitochondria are excitable organelles capable of generating and conveying electrical and calcium signals. Cell.

[81] Rizzuto R., Brini M., Murgia M., Pozzan T. (1993). Microdomains with high Ca^2+^ close to IP3 -sensitive channels that are sensed by neighboring mitochondria. Science.

[82] Szabadkai G., Bianchi K., Várnai P., De Stefani D., Wieckowski M.R., Cavagna D., Nagy A.I., Balla T., Rizzuto R. (2006). Chaperone-mediated coupling of endoplasmic reticulum and mitochondrial Ca^2+^ channels. J. Cell Biol..

[83] Feriod C.N., Gustavo Oliveira A., Guerra M.T., Nguyen L., Mitchell Richards K., Jurczak M.J., Ruan H.-B., Paulo Camporez J., Yang X., Shulman G.I. (2017). Hepatic inositol 1,4,5 trisphosphate receptor type 1 mediates fatty liver. Hepatol. Commun..

[84] Li C., Fox C.J., Master S.R., Bindokas V.P., Chodosh L.A., Thompson C.B. (2002). Bcl-X(L) affects Ca(2+) homeostasis by altering expression of inositol 1,4,5-trisphosphate receptors. Proc. Natl. Acad. Sci. USA.

[85] Bassik M.C., Scorrano L., Oakes S.A., Pozzan T., Korsmeyer S.J. (2004). Phosphorylation of BCL-2 regulates ER Ca^2+^ homeostasis and apoptosis. EMBO J..

[86] Taniai M., Grambihler A., Higuchi H., Werneburg N., Bronk S.F., Farrugia D.J., Kaufmann S.H., Gores G.J. (2004). Mcl-1 mediates tumor necrosis factor-related apoptosis-inducing ligand resistance in human cholangiocarcinoma cells. Cancer Res..

[87] Kuchay S., Giorgi C., Simoneschi D., Pagan J., Missiroli S., Saraf A., Florens L., Washburn M.P., Collazo-Lorduy A., Castillo-Martin M. (2017). PTEN counteracts FBXL2 to promote IP3R3- and Ca^2+^ -mediated apoptosis limiting tumour growth. Nature.

[88] Bononi A., Giorgi C., Patergnani S., Larson D., Verbruggen K., Tanji M., Pellegrini L., Signorato V., Olivetto F., Pastorino S. (2017). BAP1 regulates IP3R3-mediated Ca^2+^ flux to mitochondria suppressing cell transformation. Nature.

